# P-1712. Comparison of Vancomycin vs Linezolid for Treating Adult Patients with sCAP Admitted to the ICU

**DOI:** 10.1093/ofid/ofae631.1878

**Published:** 2025-01-29

**Authors:** André Emilio Viñán Garcés, Natalia Sanabria-Herrera, Sara Duque-Vallejo, Esteban Garcia-Gallo, Luis Felipe F Reyes

**Affiliations:** Unisabana Center for Translational Science, School of Medicine, Universidad de La Sabana, Chía, Colombia, Chía, Cundinamarca, Colombia; Clínica Universidad de La Sabana, Chía, Colombia, Bogota, Distrito Capital de Bogota, Colombia; ISARIC Pandemic Sciences Institute, University of Oxford, Bogota, Distrito Capital de Bogota, Colombia; Pandemic Sciences Institute, University of Oxford, Oxford, UK, Oxford, England, United Kingdom; Universidad de La Sabana, Chía, Cundinamarca, Colombia

## Abstract

**Background:**

Community-acquired pneumonia (CAP) remains the leading cause of infectious mortality worldwide. Regarding its etiology, Staphylococcus aureus has been associated with its development and a higher mortality risk than other microbiological etiologies. Current recommendations allow empiric use with either vancomycin or linezolid in patients with risk factors for MRSA, although no consensus exists on which therapy is more beneficial. This study aims to determine if there is a difference in mortality and survival between patients treated with these antibiotics.

Comparison between general characteristics of patients receiving vancomycin and linezolid.

**Figure:**
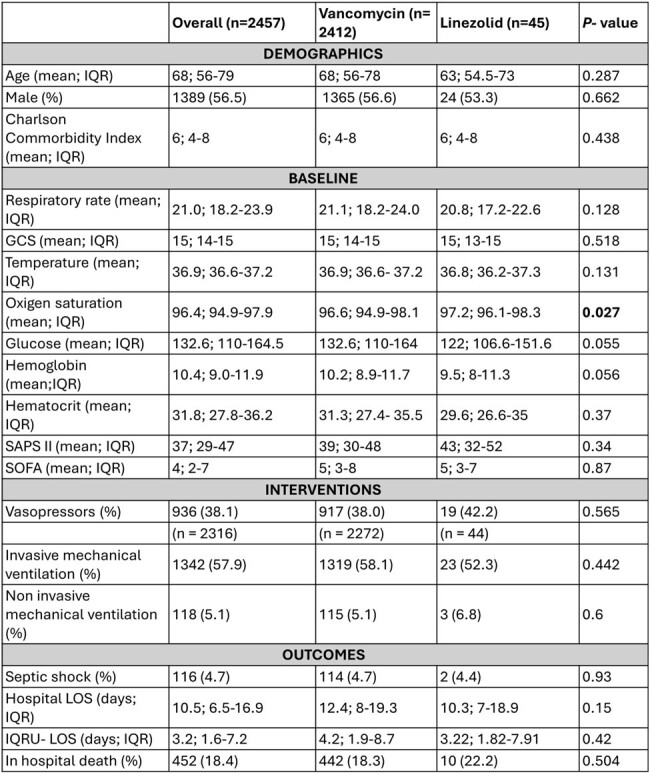

**Methods:**

Data from adult patients with CAP and ICU admission with either linezolid or vancomycin administration for at least 72 hours were extracted from the prospective MIMIC-IV database. A univariate analysis was employed to determine the differences between the patients who received each treatment. Subsequently, a survival analysis adjusted by infection severity using a Cox proportional hazards model was conducted.

Survival analysis for both in-hospital and one-year mortality. A. Kaplan-Meier for One-year Survival Probability; B. Kaplan-Meier for In-hospital 28-day mortality

**Figure:**
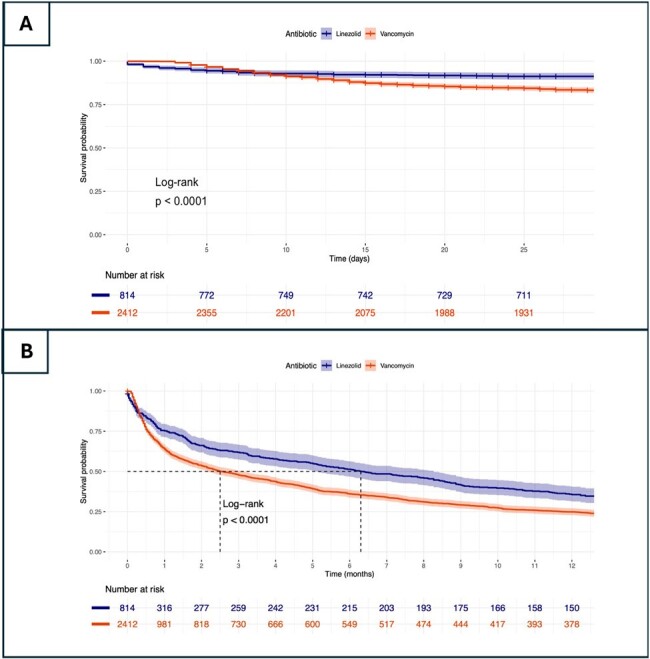

**Results:**

A total of 2457 adult patients were included, with 98% (2412/2457) receiving vancomycin and 2% (45/2457) Linezolid. The mean age was 68 (56-79), and 56.5% (1389/2457) were male. The most frequent comorbidities in both treatment groups included chronic pulmonary disease (40.9% vs 51.1%; p= 0.169) and congestive heart failure (37.9% vs 42.2%; p=0.55). Both groups presented similar severity of infection, physiological values at admission, and outcomes (Table 1). Survival analysis demonstrated a higher one-year survival probability in the patients receiving Linezolid group. Cox regression analysis revealed a significant association between SAPS II score and mortality (HR = 1.024, 95% CI: 1.02 - 1.028, p < 0.001) (Figure 1). However, when adjusting the survival analysis by disease severity, we did not find ny statistical differences.

**Conclusion:**

While there is no definitive consensus regarding the superiority of empirical anti-MRSA treatments, these results support the idea that linezolid is associated with better outcomes in terms of mortality in patients with sCAP. However, this protective effect was not observed after adjusting by disease severity. Further prospective studies are needed to support these conclusions.

**Disclosures:**

**All Authors**: No reported disclosures

